# Identifying Severity Grading of Knee Osteoarthritis from X-ray Images Using an Efficient Mixture of Deep Learning and Machine Learning Models

**DOI:** 10.3390/diagnostics12122939

**Published:** 2022-11-24

**Authors:** Sozan Mohammed Ahmed, Ramadhan J. Mstafa

**Affiliations:** 1 Department of Computer Science, Faculty of Science, University of Zakho, Duhok 42002, Iraq; 2 Department of Computer Science, College of Science, Nawroz University, Duhok 42001, Iraq

**Keywords:** transfer learning, knee osteoarthritis, hybrid learning, deep learning, SVM, X-ray, machine learning

## Abstract

Recently, many diseases have negatively impacted people’s lifestyles. Among these, knee osteoarthritis (OA) has been regarded as the primary cause of activity restriction and impairment, particularly in older people. Therefore, quick, accurate, and low-cost computer-based tools for the early prediction of knee OA patients are urgently needed. In this paper, as part of addressing this issue, we developed a new method to efficiently diagnose and classify knee osteoarthritis severity based on the X-ray images to classify knee OA in (i.e., binary and multiclass) in order to study the impact of different class-based, which has not yet been addressed in previous studies. This will provide physicians with a variety of deployment options in the future. Our proposed models are basically divided into two frameworks based on applying pre-trained convolutional neural networks (CNN) for feature extraction as well as fine-tuning the pre-trained CNN using the transfer learning (TL) method. In addition, a traditional machine learning (ML) classifier is used to exploit the enriched feature space to achieve better knee OA classification performance. In the first one, we developed five classes-based models using a proposed pre-trained CNN for feature extraction, principal component analysis (PCA) for dimensionality reduction, and support vector machine (SVM) for classification. While in the second framework, a few changes were made to the steps in the first framework, the concept of TL was used to fine-tune the proposed pre-trained CNN from the first framework to fit the two classes, three classes, and four classes-based models. The proposed models are evaluated on X-ray data, and their performance is compared with the existing state-of-the-art models. It is observed through conducted experimental analysis to demonstrate the efficacy of the proposed approach in improving the classification accuracy in both multiclass and binary class-based in the OA case study. Nonetheless, the empirical results revealed that the fewer multiclass labels used, the better performance achieved, with the binary class labels outperforming all, which reached a 90.8% accuracy rate. Furthermore, the proposed models demonstrated their contribution to early classification in the first stage of the disease to help reduce its progression and improve people’s quality of life.

## 1. Introduction

Knee osteoarthritis (OA) is a degenerative disease of the knee joint, which affects three compartments of the knee (lateral, medial, and patella-femoral) and generally develops gradually over 10 to 15 years [1,2]. Usually, it results from wear, tear, and progressive loss of articular, followed by infections that damage the joint cavity, causing discomforts such as mobility limitations, joint pain, and swelling [3]. All joints of the body are somewhat sensitive to alterations and damage to the cartilage tissue, with the knee and hip joints being more susceptible to OA due to their weight-bearing nature. Furthermore, knee OA mostly occurs in people over 55 years old, with a higher prevalence among those over 65 [4]. By the year 2050, researchers estimate that 130 million individuals worldwide will suffer from knee OA. However, early detection and treatment of knee OA help reduce its progression and improve people’s quality of life [5].

The cause of OA in the knee is not simple to detect, diagnose, or treat since it is complicated, with a relatively high number of risk variables; that is, advanced age, gender, hormonal state, body mass index (BMI) of individuals, and so on. Besides, there are other medical, environmental, and biological risk factors that are known to have a role in the development and progression of the disease, both modifiable and non-modifiable. In the worst-case scenario, patients with these risk factors undergo a total knee replacement. Currently, the only available therapies for patients suffering from knee OA are behavioral interventions, such as weight loss, physical exercise, and strengthening of joint muscles, which might provide brief pain relief while slowing the course of the disease [6,7].

**Figure 1:**
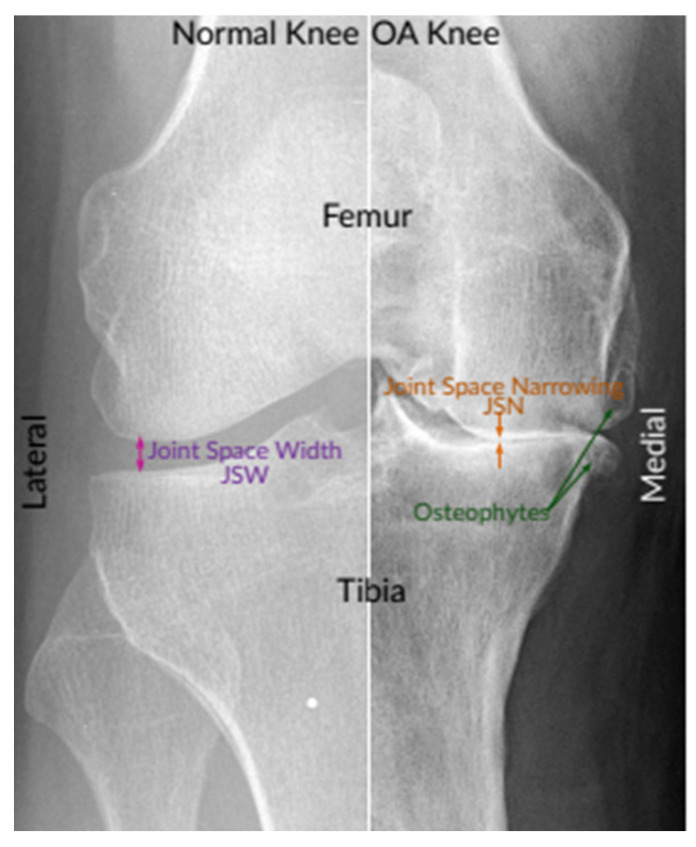
An instance of X-ray images of the normal knee and severe OA knee. On the left is a normal knee, and on the right is an OA knee. This image shows joint compartments, as well as joint space narrowing (JSN) and osteophytes [10].

Knee OA is commonly diagnosed and assessed by radiographs (X-rays), which remained the gold benchmark for knee OA screening due to its cost-effectiveness, safety, broad accessibility, and speed. According to radiologists, the most prominent pathological features of the easily observable knee OA are joint space narrowing (JSN) and osteophyte formation, as shown in Figure 1. These two features can also be used to determine the severity of knee OA using the Kellgren–Lawrence (KL) grading approach. With this approach, knee OA severity is classified depending on the consensus ground truth classification into five grades, namely, grade 0 to grade 4 [8,9].

**Figure 2:**
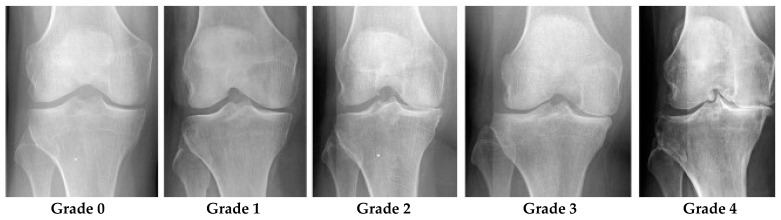
Knee joint samples of all KL grades [2].

Grade 0 denotes healthy joints in which the radiographic features of knee OA do not exist. Grade 1 denotes doubtful knee OA, which is the possibility of osteophytic lip and questionable JSN. Grade 2 denotes mild OA, which means there are clearly osteophytes as well as the possibility of JSN. Grade 3 denotes moderate OA, which means there are JSN, multiple osteophytes, and sclerosis. The last one, Grade 4, denotes severe OA because of large osteophytes in the joints marked by JSN and severe sclerosis. Figure 2 shows knee joint samples from all grades of KL [10].

With the limited number of radiologists, especially in rural areas, as well as the long time required to analyze knee X-ray images, fully automatic classification of knee severity is in great demand since it helps speed up the diagnosis process and increases the rate of early detection. For this reason, many computer-aided diagnosis (CAD)-based medical imaging approaches have been proposed in the literature to detect and analyze knee OA, such as Anifah et al. [11], Kotti et al. [12], and Wahyuningrum et al. [13].

The use of deep learning (DL) and machine learning (ML) techniques in medical imaging has recently increased in order to handle problems of classification [14,15], detection [16,17], and other associated issues without requiring a radiologist’s expertise [18].

More specifically, DL-based detection models have been designed and successfully deployed to estimate the severity of knee OA [14]. Besides, they show staggering performance in the analysis of X-rays in the biomedical domain since it does not require manual feature engineering, which takes place implicitly during the training stage by optimizing its internal parameters to fit the data of interest. Conversely, all standard ML algorithms require the given data to be transformed first using a particular feature engineering or learning algorithm to produce the desired results. Compared to standard ML algorithms, DL algorithms often require inordinate amounts of computational power and resources. Besides, it results in overfitting if fed with too little data. In addition, there are some forms of DL that yield remarkable performance in computer vision, even exceeding that of humans, such as Resnet, Inception, Xception, and CNN-based on TL [18].

Antony et al. [19] presented a novel scheme for quantifying the severity of knee OA based on X-ray images. KL grades were used as training input to train FCNN to quantify knee OA severity. Data from the Osteoarthritis Initiative (OAI) and Multicenter Osteoarthritis Study (MOST) were utilized to appraise the effectiveness of this model. Comparing the empirical results of this method to the previously existing methods revealed improvements in classification accuracy, recall, F1 score, and precision.

Norman et al. [20] proposed a novel approach for the assessment of OA in knee X-rays based on KL grading. Their approach uses state-of-the-art neural networks to implement ensemble learning for precise classification from raw X-ray images. They stated that their approach might be utilized to benefit radiologists in making a quite reliable diagnosis.

Tiulpin et al. [21] suggested an automated diagnostic technique based on deep Siamese CNNs, which acquire a similarity measure between images. This concept is not limited to simple image pair comparisons but is instead used to compare knee X-rays (with symmetrical joints). Particularly, this network can learn identical weights for both knee joints if the images are split at the central location and fed to a separate CNN branch. Simulation results on the entire OAI dataset demonstrated that their work outperforms previous models, with an accuracy score of 66.71%.

According to Chen et al. [1], two CNNs were employed to grade knee OA severity based on the KL grading system. A specialized one-stage YOLOv2 network was used to detect X-ray images of knee joints. Using the best-performing CNNs, including versions of YOLO, ResNet, VGG, DenseNet, and InceptionV3, the detected knee joint images were then classified utilizing adjusted ordinal loss analysis. Empirical results revealed that the best classification accuracy and the mean absolute error obtained with their proposed approach are 69.7% and 0.344, respectively.

Moustakidis et al. [22] developed a deep neural network (DNN)-based technique for knee OA classification, which comprises three processing steps: data preprocessing, data normalization, and a learning procedure for DNN training. Experimental results showed that their presented DNN approach was effective in improving classification accuracy.

Thomas et al. [23] proposed new deep CNNs for knee OA classification, where this model can take full radiographs as input and expect KL scores with outstanding accuracy. Based on the results reported by this study, an average F1 score of 0.70 and an accuracy of 0.71 was achieved using their proposed model.

Tiulpin et al. [3] have developed an automatic method to predict OARSI and KL grades from knee radiographs. Based on DeepCNN and leverages an ensemble network of 50 layers, and used TL from ImageNet with a fine-tuning one OAI dataset.

Brahim et al. [24] presented a computer-aided diagnostic method for early knee osteoarthritis identification utilizing knee X-ray imaging and machine-learning algorithms. Where the proposed approaches have been implemented as follows: first, preprocessing of the X-ray pictures in the Fourier domain has performed using a circular Fourier transform; then MLR (multivariate linear regression) was used to the data to decrease the variability between patients with OA and healthy participants; for feature extraction/selection stage an independent component analysis (ICA) was used for reducing the dimensionality; finally, random forest and Naive Bayes classifier were used for the classification task. Furthermore, the 1024 knee X-ray images from the public database osteoarthritis initiative were used to test this innovative image-based method (OAI).

Wang et al. [5] suggested a fully automatic scheme based on deep learning to detect knee OA using a pertained YOLO model. Based on the experimental results, their method improves knee OA classification performance compared with the previous state-of-the-art methods.

Yadav et al. [25] proposed a highly effective and low-cost hybrid SFNet. Due to the lower computation cost and high efficiency attained by training the model at two scales, the hybrid SFNet is a two-scale DL model with fewer neurons. An improved canny edge detection technique is used to locate the fractured bone first. The grey image and its corresponding canny image are then fed into a hybrid SFNet for deep feature extraction. The diagnosis of bone fractures is greatly enhanced by this process.

Lau et al. [26] developed a method based on ImageNet, the Xception model, and a dataset of X-ray images from total knee arthroplasty (TKA) patients, and the image-based ML model was created. In order to develop a clinical information-based ML model using a random forest classifier was then carried out using a different system built on a dataset with TKA patient clinical parameters. To interpret the prediction choice the model made, class activation maps were also used. The result of the precision rate and recall rate for the ML on the images loosening model reached 0.92 and 0.96, respectively, while a 96.3% accuracy rate for visualization classification was noted. However, the addition of a clinical information-based model, with a precision rate of 0.71 and recall rate of 0.20, did not further demonstrate improvement in the accuracy.

Overall, the approaches reported by the related works and the studies published in Christodoulou et al. [27], Du et al. [28], Hirvasniemi et al. [29], and Sharma et al. [30] have utilized DL and ML techniques for diagnosis different bone disease. These methods have provided excellent job performance for binary-class classification. In contrast, they are not efficient for multiclass classifying knee OA based on KL grades using X-ray images; it achieved maximum accuracy of 69% [5]. Thus, it is challenging to propose an effective tool or method for the early classification of knee OA. Therefore, an approach that uses both types of learning is crucial for improving classification performance.

In light of this, this paper aims to propose novel approaches that utilize both DL and ML algorithms to identify a given subject’s class label from a Knee X-ray image according to KL grading criteria. The proposed approaches are mainly based on two forms of learning structures, namely Deep Hybrid Learning-I (DHL-I) and Deep Hybrid Learning-II (DHL-II). The first one, DHL-I, is based on CNN structure, in which a new structure of five classes of prediction was developed to be initially trained on knee X-ray images and then used as feature extraction. A PCA was then applied to reduce these learned features and then fed into SVMs, which can classify knee OA by pattern discrimination. Whereas the second one, DHL-II, is the same as the first one except for the following. The pre-trained CNN developed for the DHL-I was fine-tuned using the TL concept to classify knee OA into four classes, three classes, and two class labels.

The primary motivation of this study is to build up a strategy that mixes ML and DL methods to develop an efficient DHL to classify knee OA severity based on KL grades in different categories. To examine how different class-based classifications (i.e., binary and multiclass) affect prediction performance. As a result of this process, knee OA severity classification is significantly improved on the side of saving time, accuracy, and hardware costs, and these may contribute to early classification in the first stage of the disease to help reduce its progression and improve people’s quality of life.

The following are the main contributions of this paper:

- To the best of our knowledge, DL techniques have not yet been used for feature extraction purposes in the literature on knee OA. Thus, the proposed model is the first to show their potential application in this area.
- Unlike the existing studies that develop a classification model for specific n-class labels, this paper proposes various classification models for classifying the severity of knee OA, i.e., five classes, four classes, three classes, and two classes model-based.
- Despite many articles on TL and fine-tuning, as far as we know, no research has compared or assessed the two approaches in terms of pre-trained deep feature classifications for knee OA.
- This model combines deep and hand-crafted features obtained by a proposed pre-trained CNN and the PCA algorithm to generate the most prominent feature set before being sent to the SVM algorithm for classification.
- The concept of TL was employed to fine-tune the proposed pre-trained CNN developed on five classes’ labels to fit other class labels, i.e., four classes, three classes, and two classes model-based.
- As compared with the existing state-of-the-art methods for predicting knee OA, the proposed DHL models performs significantly better.

The rest of the paper is arranged as follows. The theoretical foundation is presented in Section 2, and the materials and techniques employed in this work are described in Section 3. Section 4 presents the experimental results and their explanations. Finally, Section 5 brings the paper to a conclusion and discussion.

## 2. Theoretical Background

A brief theoretical background regarding the methods used by the proposed approach is presented in the following subsections to assist the reader in becoming familiar with those techniques.

### 2.1. Convolutional Neural Networks (CNNs)

One of the most famous and commonly used algorithms in DL is CNN. Which has the main benefit over its predecessors of automatically identifying the relevant features without human intervention. It is inspired by the human brain’s visual system. As a result, CNNs are designed to enable computers to see the world in accordance with human perception. Natural language processing, image classification, and image recognition can all be performed using CNNs in this way [31]. The CNN is a type of DNN that often contains a convolutional layer, activation layer (a nonlinear activation layer), pooling layer, fully-connected layer, and output layer [32,33]. The following are the CNN layers:

#### 2.1.1. Convolutional Layer

The convolutional layer is the main layer of a CNN, which performs an operation called “convolution” which gives CNN its name. Furthermore, kernel convolution is an essential part of several other computer vision technologies. It plays a significant role in how CNN operates, which consists of several convolutional filters called kernels. It is a method in which we apply a small number matrix to our image known as a kernel or filter, then transform it using the values of the filter [34].

Moreover, each filter corresponds to a specific matrix that performs the convolution operation on the input image. The input image is convolved with these filters to produce the output feature maps. For instance, this formula is used to compute feature map values, as shown by Equation (1).

(1) $$F\left[m,n\right]=\left(I*K\right)\left[m,n\right]=\underset{i}{\sum }\underset{j}{\sum }K\left[i,j\right]I\left[m-i,n-j\right]$$

where *I* represents the input image and *K* is the kernel. The row and column indexes of the result matrix are represented by *m* and *n*, respectively. Using a convolutional method, we produced an output feature map. Each output feature map in the convolution layer is combined with numerous input feature maps, as seen in Equation (2). The presented model consists of five convolution layers (detailed in Section 3.4.1).

(2) $$X_{b}^{a}=I\left(\underset{i\in M\;b}{\sum }X_{b}^{a-1}*K_{ij}^{a}+b_{b}^{a}\right)$$

where, $X_{b}^{a}$ is the output of the present layer, $X_{b}^{a-1}$ is the previous layer’s output, $K_{ij}^{a}$ is the current layer’s kernel, $b_{b}^{a}$ are the current layer’s biases, and M represents a collection of input maps. After that, the convolution results are processed by a nonlinear activation function.

#### 2.1.2. Activation Layer

The activation layer (or nonlinear layer) follows each convolution layer immediately. The objective of this layer is to provide nonlinearity to a system that mostly computes linear operations. For instance, the rectified linear unit (ReLU) activation layer with a convolutional layer allows for increased nonlinearity in the input data, which converts the whole input values into positive numbers due to the fact that the ReLU is 0 for all negative inputs. Additionally, the main advantage of ReLU over other activation functions is that it reduces the computational load [35,36]. Therefore, CNN with ReLU is faster and easier in the current scenario. It can be expressed mathematically by Equation (3);

(3) f(j)=max(0,j)

Here, the function implies that the output f (j) is zero for all negative values and the output for positive values remains constant.

#### 2.1.3. Pooling Layer

**Figure 3:**
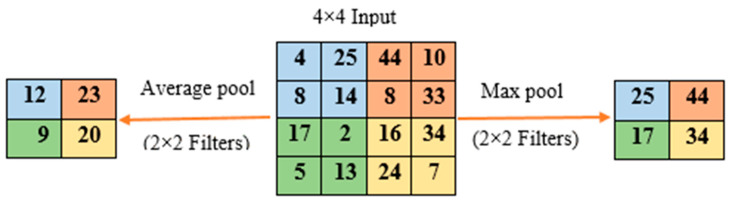
Two types of pooling operations.

Convolution layers are often interleaved with subsampling or pooling layers in CNN architectures. Based on the convolution layer, each feature map is processed independently. In order to minimize overfitting and the number of extracted features, pooling operations reduce the spatial size of the feature map. A maximum pooling method and an average pooling method are the most common pooling methods [37]. The two methods of pooling are shown in Figure 3.

#### 2.1.4. Fully Connected Layer

Even though the output of the convolution layer and the pooling layer allows us to extract high-level features of the input images, combining fully connected (FC) layers may be much more advantageous, since adding an FC layer is also a low-cost way to learn the nonlinear combinations of these attributes. The FC layer produces a promising classification score for labeling the input images in the input layer by combining convolution and pooling layers. Then, the FC layer sends the two-dimensional result to the output layer, where a sigmoid function or softmax can be used for predicting the input class label [38]. A softmax and sigmoid activation function can be demonstrated mathematically by Equations (4) and (5), respectively;

(4) $$\sigma {\left(x\right)}_{j}=\frac{e^{x_{j}}}{\sum_{k=1}^{k}e^{x_{k}}}$$

(5) $$f\left(y\right)=\frac{1}{1+e^{-y}}$$

Here, *x* and *y* represent the values from the neurons of the output layer, σ(x)  can be thought of as the predicted probability of the test input belonging to class *j*, and *k* is the amount of output classes in multiclass classification. Additionally, the CNN model uses the loss function to calculate how distant an estimated value is from the true value. It helps to define what a good prediction would be for the model. The loss function for classification problems is the cross-entropy loss function, which is formalized in Equation (6). Where, for binary classification, the cross-entropy loss function can be described in Equation (7):

(6) $$L\left({\hat{y}}_{j},y_{j}\right)=-\overset{M}{\underset{j=1}{\sum }}y_{j}\left(M\right)\log{\hat{y}}_{j}\left(M\right)\;$$

(7) $$LB\left({\hat{y}}_{j},y_{j}\right)=-\frac{1}{M}\overset{M}{\underset{j=1}{\sum }}[y_{j}\log{\hat{y}}_{j}+\left(1-y_{j}\right)\log\left(1-{\hat{y}}_{j}\right)]\;$$

where *M* is the training dataset size, ${\hat{y}}_{j}$ is the predicted output label and *y_j_* is the actual output label.

### 2.2. Deep Transfer Learning Techniques

TL is a technique for obtaining deep features from pre-trained CNN models [39]. Using TL in computer vision is time-saving because it allows us to build an accurate way of developing a model by using a model that has been trained for one problem as a starting point for a second related problem. Computer vision and natural language processing problems are typically solved using pre-trained models as the basis for model parameters for deep learning. Thus, it is easy for the algorithm to adjust the weights of the training set in accordance with the new domain by slightly altering them. A classification model, for example (radiographs), is based on pre-trained models from very large datasets within computer vision, such as ImageNet. Therefore, TL works by using patterns learned by solving various problems instead of starting from scratch [39,40]. As a result, you avoid having to start over from scratch. When employing TL on computer vision issues, two methodologies are often used [40]: Firstly, homogenous transfer learning is used whenever the source domain and the target domain share the same feature space. This type of TL is used in the proposed model. Secondly, heterogeneous transfer learning occurs when the source and target do not have similar or identical feature spaces.

## 3. Materials and Methods

In this section, the materials and methods used to develop the proposed model are described. In Section 3.1, the datasets of X-ray images are described, along with their features and classes. The next step will be the preprocessing of the data in Section 3.2. The validation schema will be presented in Section 3.3. In Section 3.4, the proposed framework will be presented, and in Section 3.5, the proposed performance metric will be described.

### 3.1. Dataset

**Figure 4:**
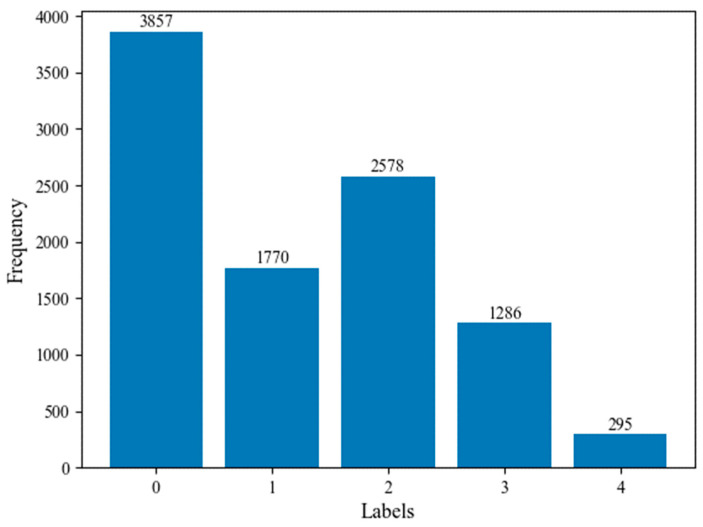
The distribution of instances across the KL grading system for assessing knee OA severity.

We used a publicly accessible dataset of knee X-rays: Osteoarthritis Initiative (OAI) (https://nda.nih.gov/oai/ (accessed on 8 October 2021)) to conduct the experiments examined in the proposed model. This study examined knee osteoarthritis in multiple centers over a long period of time. As determined by the OAI coordinating center at UC San Francisco, 4796 people aged 45 to 79 were enrolled by using the original dataset with KL grades for both knees. In addition, the data include bilateral posterior-anterior PA fixed flexion of knee X-ray images from the OAI. Figure 4 illustrates the distribution of instances across the KL grading system for assessing knee OA severity. In addition, this study uses X-rays of the knee in order to perceive KL grades from one X-ray image of both knee joints, we keep only those X-ray images with KL grades available on both knee joints. In total, 9786 X-ray images with knee joints were applied to appraise the effectiveness of our proposed model. The reason behind choosing this dataset is that it is one of the most extensively used datasets for knee osteoarthritis prediction, as evidenced by previously published studies.

### 3.2. Data Preprocessing

**Figure 5:**
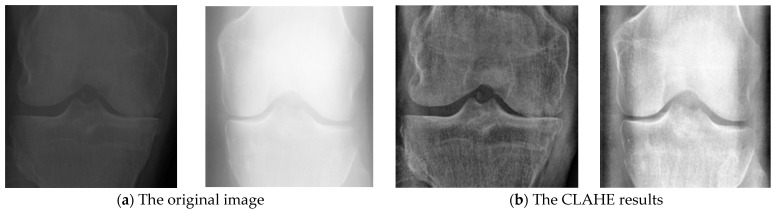
Applying CLAHE process on dataset images. (**a**) shows the original image, and (**b**) illustrated the result images after the CLAHE has been applied.

We implemented two types of data preprocessing on the images. As such, we first resize the images from (224, 224) into a fixed mean size of (112, 112), which is considered a critical preprocessing step in computer vision. Since the machine learning models train the model faster on smaller images size. Usually, the quality of an image has an impact on the performance of the overall algorithm. Therefore, contrast-limited adaptive histogram equalization (CLAHE) was used in the preprocessing method to improve the radiograph’s contrast in order to make an accurate classification. Mapping CLAHE gives the input image intensity values in such a manner that the histogram of the generated images has a fairly uniform distribution, where it employs two essential parameters, namely the number of tiles and the clip limit. Hence, CLAHE can have a large impact on the results if the parameters are adjusted. Indeed, these parameters determine the efficiency of CLAHE. Therefore, the optimal parameter values that the proposed model depends on are clip limit = 5.0, and tiles = (8, 8). According to the experiments, CLAHE normalized the whole image so that it can be further processed. As a result, images were so varied that they were difficult to process without CLAHE. Figure 5 shows the results of knee OA, where Figure 5a contains the original image, whereas Figure 5b represents the CLAHE results.

### 3.3. Strategy of Validation

There are several forms to validate a developed machine learning algorithm, such as k-Fold cross-validation, hold-out, or even manual assessment from experts. Of these, the hold-out is the most adopted one when dealing with a large dataset. Therefore, in this study, the hold-out strategy was used to randomly partition the OAI dataset into a training set (80%) and a testing set (20%). Ten percent of the training data was used for validation during the training process to ensure the generalizability of model performances. Table 1 shows the frequency distribution among training, validation, and testing sets based on KL grades. Moreover, we selected this data division technique since it is consistent with most previous studies’ approaches and allows us to compare our approach to theirs fairly.

### 3.4. The Proposed Framework

**Figure 6:**
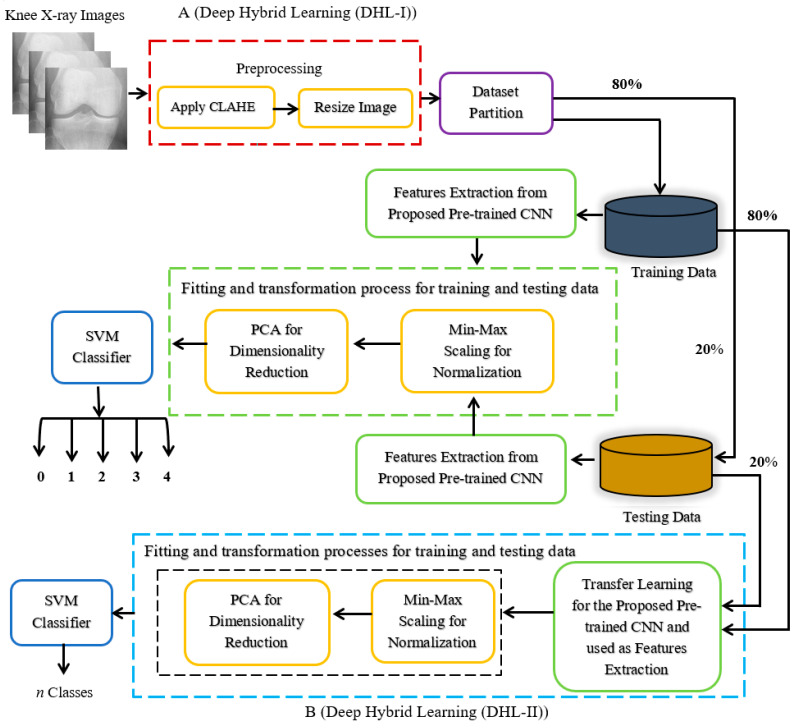
The framework of the proposed approach. Part (**A**), DHL-I presents a proposed model for knee OA classifications using KL grade, while part (**B**), DHL-II provides a detailed overview of the proposed knee OA classifications based on n-class labels.

The framework of the proposed approach is shown in Figure 6. First, the given dataset was preprocessed and split into training and test sets, as explained in Section 3.2 and Section 3.3. Then, unlike other existing research that develops a classification model for a specific *n* classes category, we developed a classification model for each *n* classes category to determine the overall performance of the proposed models; that is, two classes, three classes, four classes, and finally five classes-based models. Primarily, the developed models fall into two frameworks: DHL-I and DHL-II.

The proposed DHL-I makes use of three techniques: CNNs, PCA, and SVM. Here, a particular pre-trained CNN structure was used to extract salient features from X-ray images. Then, the PCA algorithm was applied to the extracted features for dimensionality reduction. Lastly, the SVM classifier was used to classify the knee OA into five classes according to the KL grading criteria, whereas the proposed DHL-II uses the concept of TL to develop three models, i.e., four classes, three classes, and two classes, from DHL-I by fine-tuning the pre-trained CNN model to fit the data of different *n* classes labels. The following subsections will discuss the procedure in detail.

#### 3.4.1. CNN Model Architecture

In this section, we briefly describe the proposed CNN, which consists of an input layer with (112 × 112 × 1) dimension; the network also contains mainly six layers of learned weight’s structure: five 2-dimensional convolutional layers (2_dConv) and one FC layer. The first block contains three 2_dConv layers that have a filter size of 32 with a (3, 3) kernel size. It is followed by the max-pooling layer with pool size (2, 2), while the second block is another 2_dConv, with kernel size (3, 3) and 64 filter size. The third block involved 2_dConv, the kernel size is (3, 3) with a filter size of 128, which is followed by the average pooling (Avg pool) layer with pool size (2, 2). Based on our architecture, we used the Avg pool at the end of each last two-block; due to maintaining the higher resolution assists the system comprehends further local features to support diagnosing knee OA since these are relevant for diagnosis. Then, the convolution feature map is converted into a one dimension flatten process, which organizes all of the feature data generated by convolutional layers output into a single vector. After flattening, the vector data is fed to the next levels of the CNN, known as FC layers or dense layers. In order to assign random weights to inputs and predict the appropriate label after doing feature analysis and computation. The last stage of the CNN is to inspect the process of classification of knee OA severity based on KL grades 0, 1, 2, 3, or 4 by using the Softmax activation function to generate their class probabilities.

**Figure 7:**
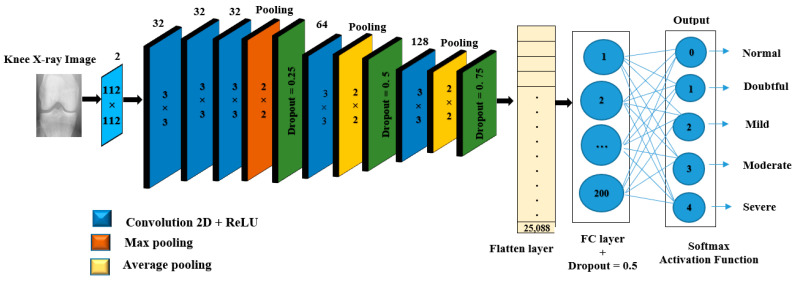
Proposed CNN architecture.

Furthermore, the whole network uses the Relu activation function, which determines whether neurons fire or not, with padding the same in convolutional layers and dropout after each pooling layer can help avoid over-fitting and an FC layer with different parameters. Figure 7 and Table 2 illustrate the detailed architecture of the proposed CNN. Indeed, the reason behind this architecture is that the diagnostic problem of knee OA with plain radiographs is time-consuming and laborious because these regions are very complicated, which is considered a fine-grained problem. Thus, we designed the network using 2_dConv with five layers, each reducing the input size, in order to keep a strong signal from the first layer.

The models were trained from scratch utilizing categorical cross-entropy considering the KL grades as the ground truth. In addition, the network weights were adapted with the Adam algorithm, which is a stochastic gradient descent algorithm with a learning rate (LR) = 0.001, β1 = 0.9, β2 = 0.999, and batch size of 256, whereas in our case, we used 50 epochs. The details of the CNN approach and its mathematical representation are described in Section 2.1.

##### Deep Feature Extraction

Once the training process of the proposed CNN was completed, the output layer was removed, and then the deep features were extracted to obtain deep information to enhance generalization performance. The pre-trained CNN model was comprised of three feature extraction blocks. In each block, there were two 2_dConv layers, as shown in Equation (8), coupled with a ReLU activation function to utilize spatial correlation and address nonlinearity in the dataset. In addition, to further extract features associated with area homogeneity and edges, max-pooling *X_max_* and average *X_avg_* were accomplished after each block, which are described by Equations (9) and (10), respectively [41]:

(8) $$h_{i,j}=f\left(\overset{m}{\underset{p}{\sum }}\overset{m}{\underset{q}{\sum }}W_{p,q}.X_{i+p-1,j+q-1}+b_{p,q}\right)$$

(9) $$X_{avg_{i,j}}=\frac{1}{K^{2}}\overset{K}{\underset{a=1}{\sum }}\overset{K}{\underset{b=1}{\sum }}h_{i+a,j+b-1\;}$$

(10) $$X_{max_{i,j}}=max_{a=1,\ldots ,k,b=1,\ldots ,k}h_{i+a,j+b-1}$$

**Figure 8:**
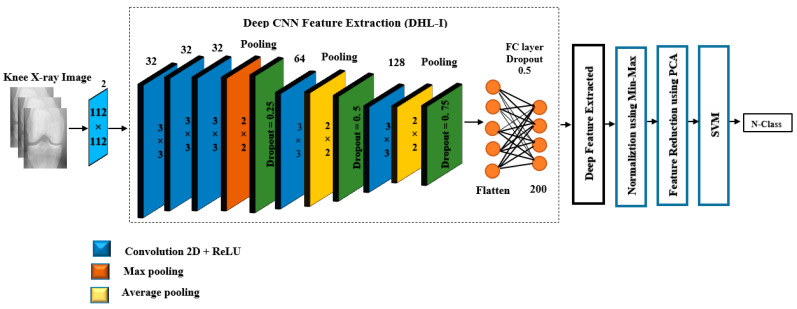
The proposed DHL-I for knee OA classification, based on KL grades.

In Equation (8), the two-dimensional kernel represents $W_{p,q}$ of size (*p*, *q*) and Χ denotes the input, *m* indicates the kernel width and height, *b* represents the bias units, $h_{ij}$ refers to convolution output, and f(.) as a transfer function. *K* stands for the window dimension of the average and max pooling, as shown in Equations (9) and (10)**.** In these regards, we generated 200 deep features from the second last flattened layer of the developed CNN model. Figure 8 illustrates the systemic process after the feature was extracted.

##### Deep Hybrid Learning-I (DHL-I)

After extracting 200 features from the pre-trained CNN, a MinMax scaler was applied to them. The purpose of this was to normalize the data within a specific range, usually between (0–1), without changing the shape of the original distribution. In addition, it speeds up calculations in an algorithm and increases model accuracy. In this method, each feature was scaled to a range with formulas in Equation (11);

(11) $$X_{scaled}=\frac{X-X_{min}}{X_{max}-X_{min}}$$

Normalized values are denoted by *X*, while feature ranges are indicated by (*X_min_*, *X_max_*). Moreover, the normalized features are assigned to the PCA method. It is considered a technique to reduce the dimensionality of data and reveal information that can be used to make decisions and improve machine learning effectiveness. Furthermore, it plays a crucial role in experiments under illumination since it minimizes the effect of noise. In the PCA algorithm, the covariance matrix (A) was initially computed from the normalized features. The covariance matrix was then decomposed into singular values to determine the principal components (PCs). Thereafter, the eigenvectors and eigenvalues are computed, as shown in Equations (12) and (13). We need to calculate eigenvectors and eigenvalues from the covariance matrix in order to determine the PCs of the data. Where *I* is the identity matrix of the same dimension as *A,* and *det* is the determinant of the matrix. The eigenvalue in PCA represents its magnitude, while the eigenvector determines the direction of maximum variance [42].

(12) (A−λΙ)=0

(13) det(A−λΙ)=0

**Figure 9:**
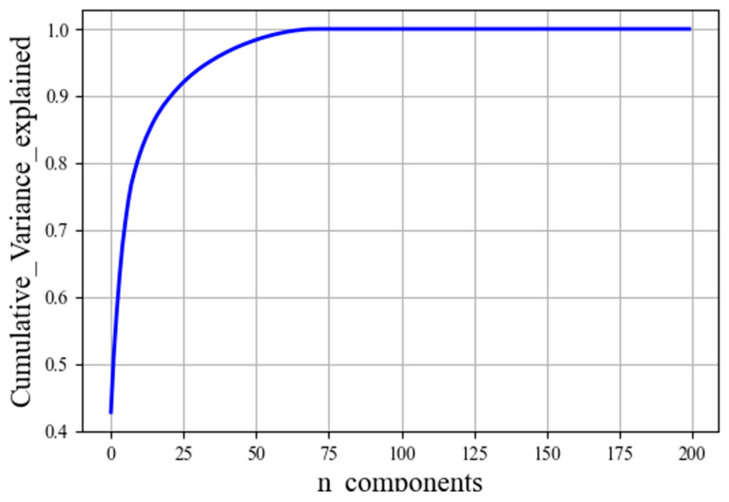
The explained variance for all the components.

Figure 9 illustrates how well PCA captures the explained_variance_ratio among the data. It is evident from Figure 9 that there are 200 dimensions in our dataset, therefore, the variance is explained by 200 principal components at 100%. The first component alone captures approximately 42.79% of the variability in the dataset, and the second component alone captures approximately 51.44% of the variability in the dataset, etc. According to this study, we set the number of components to 0.99. This will select the number of components while preserving 99% of the variability in the data. In this case, the algorithm has found 15 principal components that preserve 99% of the variability of the data.

Finally, the classification process is the final stage of our proposed model, the components that are predicted to capture the high variance are chosen and then fed to SVM in order to categorize knee OA severity based on KL grades. The SVM is a set of supervised learning methods that are used to detect outliers and perform classification and regressions. Essentially, SVMs classify linearly separable data, however, the feature vectors might not be linearly separable. Therefore, the SVM classifier is dependent on the kernel function to overcome this problem, such as (polynomial, radial basis function, and sigmoid) [43]. In this study, the radial basis function (rbf) has been employed since it achieved high performance than other kernel functions, where it is mathematically represented in Equation (14):

(14) $$Κ\left(x_{a},x_{b}\right)=exp\left(\frac{∥x_{a}-x_{b}{∥}^{2}}{2\sigma^{2}}\right)$$

In Equation (14), the reduced feature is referred to as *x*, whereas the value of σ affects the transformation of data; changing its value controls the performance of the SVM. Besides, SVM constructs an optimal hyper-plane to get an optimal model by selecting the appropriate values of gamma and C parameters in order to train the model. C is a penalty parameter or regularization constant and provides balancing of the two conflicting criteria, namely margin maximization and error minimization. Gamma is a parameter that defines where the influence of a single training example reaches, with low values meaning “far” and high values meaning “close”. In the proposed model, we used C = 1000 and gamma = 0.001.

#### 3.4.2. Transfer Deep Model for Features Learning and Extraction

We adopted a TL approach in the proposed pre-trained CNN for knee OA classification based on KL grades. The full details of the pre-trained network utilized, with their input size, the number of layers, as well as the number of parameters, are illustrated in Figure 7 and Table 2. For all experiments (with and without TL from the X-ray knee images), the same training strategy was utilized. The idea is to perform a TL experiment to train a deep network by applying a fine-tuning process to estimate the stage of knee OA in three cases (four classes, three classes, and two classes). The modification consists of increasing the number of FC layers.

**Figure 10:**
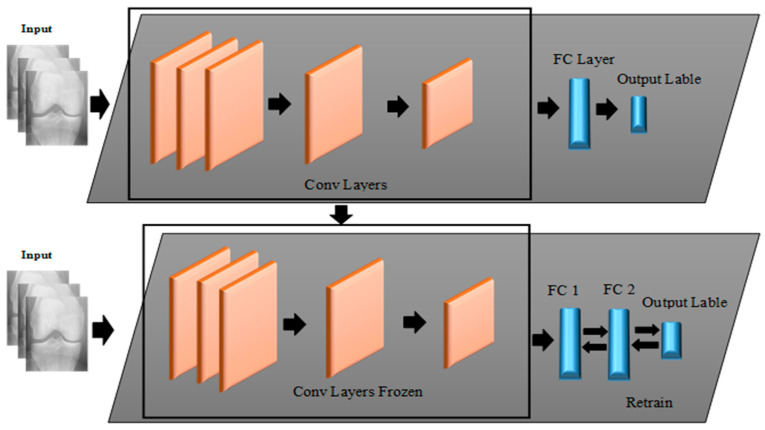
The block diagram of deep modeling for the fine-tuning transfer learning.

Implementing TL involves the following steps: Firstly, the three convolutional blocks which were used during the training for knee OA are reused in the modified CNN; these convolutional layers parameters were frozen, as shown in Figure 10. Assuring that the extracting knee features are unchanged by freezing the training process on the convolutional layers. Secondly, the top FC layer of the pre-trained model is removed and replaced by three new FC layers of different sizes. The sizes of the first two FC layers are acknowledged as 512 and 256, respectively. The first FC is followed by a dropout layer with 0.5 sizes. The last FC layer calculates the probability of each label based on a softmax activation, which represents the number of labels in the OAI database, and replaces it with a new softmax layer relevant to the problem. However, sigmoid activation was used in the binary classification problem. As a result, all learning is carried out solely on the FC layers in order to identify the classes.

In order to train the models for multiclass and binary classification, categorical cross-entropy and binary cross-entropy, respectively, were used by applying the Adam optimizer to train the modified CNN to find the optimal parameters for better classification accuracy with LR = 0.001, β1 = 0.9, β2 = 0.999, and batch size of 256 for 20 epochs. This modification on the CNN is performed three times to obtain three pre-trained models to classify knee OA grades into 4, 3, and 2 categories. Having trained the models, they are used for feature extraction, followed by the same process as mentioned in Section 3.4.1 until they reach the classification stage, as shown in Figure 6B.

### 3.5. Evaluation Metrics

This section illustrates the evaluation parameters that are used in the proposed scheme. The knee OA classifier models were assessed using standard performance metrics, which consist of sensitivity, often referred to as recall, accuracy, specificity, precision, and F-measure [44]. Firstly, accuracy is the ratio of properly categorized cases to the total number. Thus, it calculates to assess the overall performance of the given approach on the data and is represented mathematically by Equation (15):

(15) $$Accuracy=\frac{\mathrm{TP}+\mathrm{TN}}{\mathrm{TP}+\mathrm{TN}+\mathrm{FP}+\mathrm{FN}}$$

TP (true positive) refers to cases that are accurately classified as positive, FN (false negative) refers to how many positive cases were wrongly classified as negative, FP (false positive) indicates instances that are incorrectly classified as positive, and TN (true negative) indicates the negative cases that were correctly classified as negative.

Secondly, sensitivity (recall) in Equation (16) predicts the probability of finding all positive units in the dataset. Therefore, it is the ratio of patients who are accurately predicted over all relevant occurrences.

Thirdly, Precision in Equation (17) is the ratio of correctly predicted positive categories to all elements that are expected to be positive, while, specificity in Equation (18) is used to measure the accuracy of the proposed model in predicting the class that needs to declare as the negative class. Finally, the F-measure, also called F-score in Equation (19), incorporates precision and recall. As a result, the harmonic mean (average) is calculated from both precision and recall:

(16) $$\mathrm{Sensitivity}/\mathrm{Recall}\left(S\right)=\frac{\mathrm{TP}}{\mathrm{TP}+\mathrm{FN}}\;$$

(17) $$\mathrm{Precision}\;=\frac{\mathrm{TP}}{\mathrm{TP}+\mathrm{FP}}\;$$

(18) $$\mathrm{Specificity}=\frac{\mathrm{TN}}{\mathrm{TN}+\mathrm{FP}}$$

(19) $$F-\mathrm{measure}\;=\frac{\left(2\times \mathrm{Precision}\times \mathrm{Sensitivity}\right)}{\left(\mathrm{Precision}+\mathrm{Sensitivity}\right)}$$

Additionally, to ensure the performance of the proposed model, we used other metrics including receiver operator characteristic (ROC), which is a probabilistic curve that plots the TPR (true positive rate) versus the FPR (false positive rate) at different thresholds. To judge whether a model can distinguish between classes, it is evaluated using the area under the curve (AUC). At different thresholds between positive and negative classification, models with higher AUCs perform better. ROC curves have an AUC ranging from 0.5 to 1, where 0.5 ≤ AUC ≤ 1. As a result, a classifier with an AUC of 1 is able to distinguish between all positive and negative class points correctly. Nonetheless, when AUC is 0, all negatives are predicted as positive and vice versa. Similarly, the classifier is unable to discriminate between the positive and negative classes when AUC equals 0.5 [45].

## 4. Experimental Results

This section reports the experimental results for all models. The models were designed and simulated using Tensorflow in Python 3.8 on a computer equipped with an Intel(R) Core (TM) i5-2450M CPU at 2.50GHz and 8 GB of RAM. In addition to these elements, we used a Samsung 256 GB SSD, a GeForce GT 610M graphics card with 2 GB, and a 64-bit Windows 10 to train the models. This study provides a method for diagnosing and grading knee OA based on plain radiographs. Utilizing particular disease-related features similar to those considered in clinical care (e.g., bone shape, joint space, etc.). Therefore, in order to identify the effectiveness of the model, we used unseen (testing) data, which consisted of 1958 samples of knee X-ray images selected at random. Furthermore, for each experiment conducted, the same dataset was used, and the same splitting method was applied as in Section 3.3. Moreover, various evaluation metrics were utilized to evaluate the proposed approach, as summarized in Section 3.5.

### 4.1. Experiment 1: Assessing the Performance of the Proposed CNN Knee OA Severity Using CNNs

**Figure 11:**
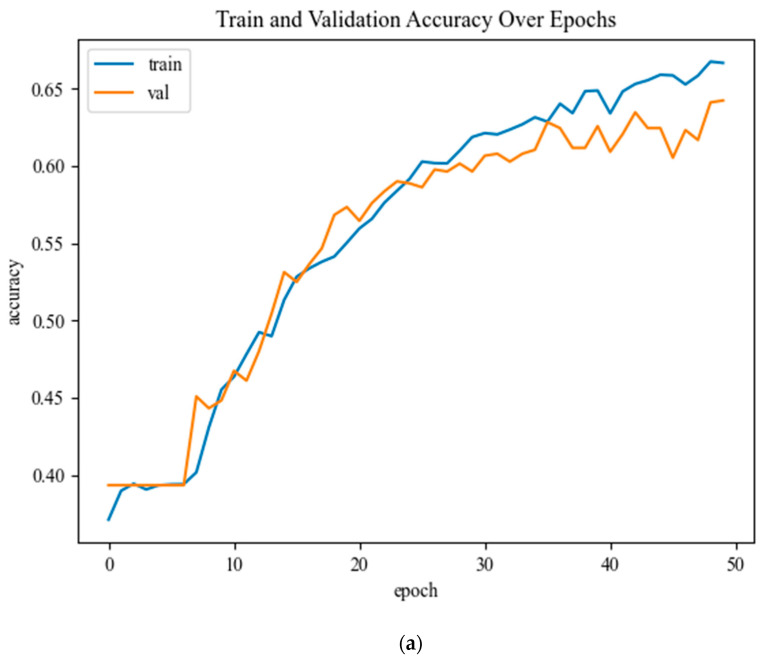

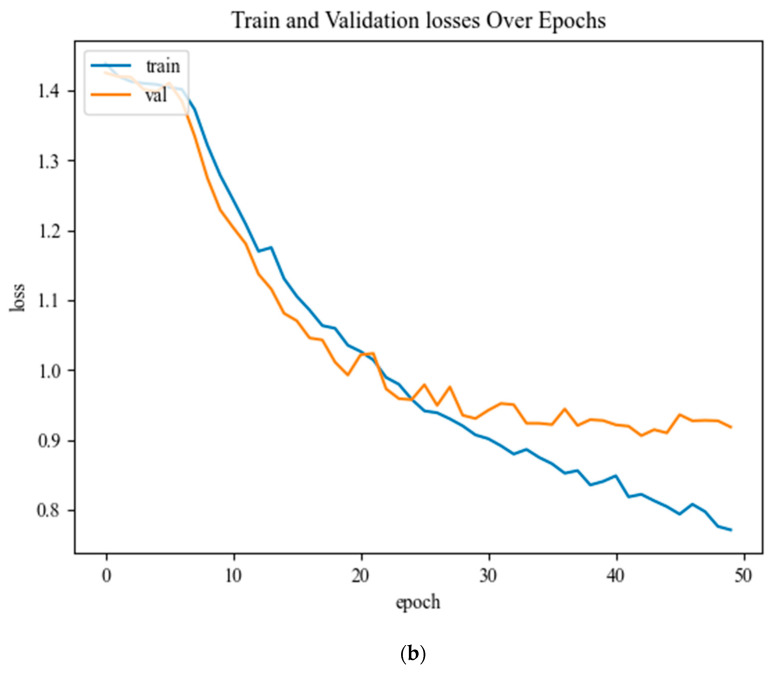
Model performance (**a**) displays the training and validation of accuracy, and (**b**) shows the loss in each period.

This section summarizes the experimental results of the proposed CNN architectures that were trained from scratch. The model has an accuracy of 62% in testing data, an average recall of 59%, an average precision of 63%, an average specificity of 88.7%, and an average F1 score of 59.6%. The model is considered to be the best based on the fact that the training and validation data were not overfitted or under fitted. Based on the history of the model for the training and validation losses and accuracy over epochs in Figure 11, the accuracy of training and validation was analyzed. Overfitting is evident when a large difference exists between training and validation. It is important that the validation is equal to or marginally less accurate than the preparation to produce the best model. On the plot of accuracy, the model could have been trained a little more since both datasets show rising accuracy over the past few epochs, while loss decreases with each epoch.

**Figure 12:**
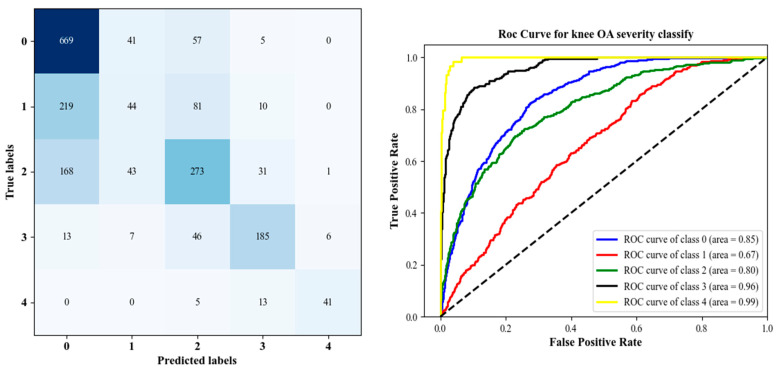
Left is the confusion matrix, and right shows the ROC curve results using the proposed CNN model.

As a further confirmation of the CNN model’s effectiveness, Figure 12 provides its ROC curves, AUCs, and confusion matrix. A confusion matrix and other metrics suggest that classifying Knee OA images conditioned on KL grade 1 is challenging because of the small variations, particularly between grades 0 to grade 2. As a result, this affected the rate of accuracy of the model in recognizing these classes as described in Table 3.

### 4.2. Experiment 2: The Proposed DHL-I Performance Based on Feature Extraction for Classifying Knee OA Severity Using Pre-Trained CNNs

This experiment used the presented pre-trained CNN obtained from the previous experiment to extract features by simply eliminating its output layer. As there were 200 features extracted from the pre-trained CNN, we used the PCA method to reduce their dimensionality. The PCA features were then provided to SVM to classify knee OA severity into five classes. Therefore, the ultimate knee OA prediction method was developed, known as DHL-I. Here, we found a significant difference observed between the proposed CNN model and DHL-I. Specifically, a performance of 74.57% accuracy rate for the average test set was achieved on the DHL-I approach for classifying knee OA depending on KL grades, whereas the knee OA classification accuracy of the CNN model was 62%. Furthermore, Table 4 provides detailed comparisons of standard performance measures, including sensitivity, F-score, specificity, and accuracy. The table also shows that combining DL with ML results in higher performance rates for each measure compared to those achieved by CNN models alone. According to the results, average recall, average precision, average specificity, and average F1-coefficient were 75.4%, 76.8%, 92.5%, and 74.8%, respectively.

**Figure 13:**
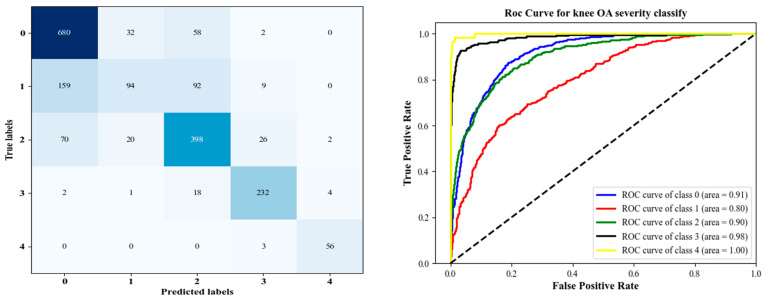
The confusion matrix for KL grading is shown on the left, and the Roc curve of SVM with 99% PCA components produced from 200 features is shown on the right.

In addition to the metric-based evaluation, the ROC curve and confusion matrix were also used to assess the performance of a classifier to separate infected from uninfected samples. Figure 13 illustrates the ROC curve and confusion matrix. Based on the empirical evaluation, it is evident that the proposed framework outperforms the previous network trained only for classification. According to the grading confusion matrix obtained by applying the proposed method to the confusion matrix items, the number of correct classifiers outperforms the number of misclassifies, but classification metrics for grade 1 remain low. On the other hand, we can notice that classifying from grades 0 to 2 is improved compared to the proposed CNN. In addition, as shown in Figure 12 and Figure 13, the ROC curve (KL > 2) achieved an AUC greater than 0.95, which is higher than any previously reported value. Generally, the network trained from scratch did not perform better than the network trained from DHL-I.

### 4.3. Experiment 3: The Performance of TL and Feature Extraction for Classifying Knee OA Severity Using Different Classes

We conducted additional evaluations of the OAI dataset’s strata by making some changes in the strata based on the related research. This was made using pre-trained CNN architectures with fine-tuning to explore how fine-tuning each block affects generalizability. Thus, three pre-trained CNNs will be built for classifying knee OA at different stages. As a result, each of the three models has been used independently to extract features until they reach the classification process illustrated in Section 3.4.1 for classifying knee OA into four classes, three classes, and two classes based on the three strata described in the following subsection, in order to develop a new version of DHL-II to predict knee OA.

**Figure 14:**
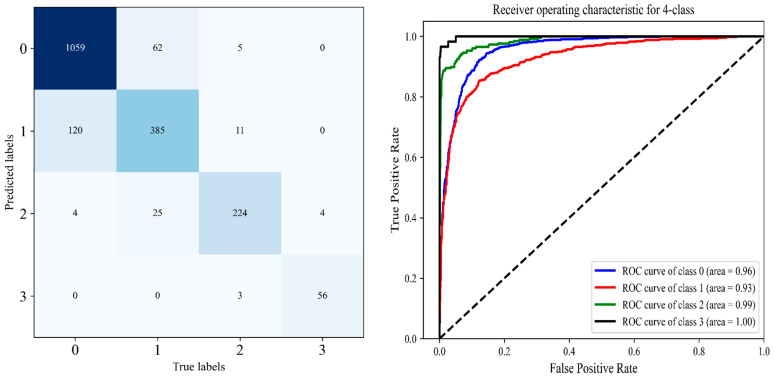
Left is the confusion matrix, and right shows the ROC curve obtained from DHL-II for classifying knee OA using four classes.

**Figure 15:**
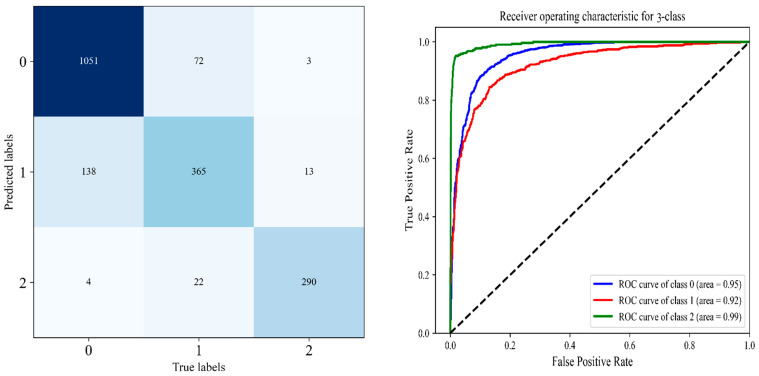
Left is the confusion matrix, and right shows the ROC curve obtained from DHL-II for classifying knee OA using three classes.

**Figure 16:**
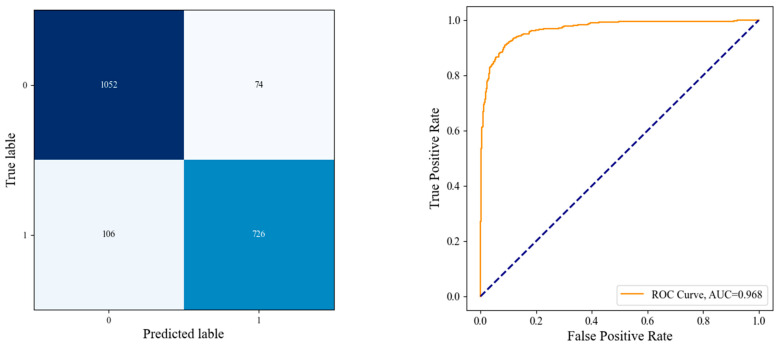
Left is the confusion matrix, and right illustrates the ROC curve obtained from DHL-II for classifying knee OA using two classes.

We observed a large improvement in the performance of these models as compared with the other models due to freezing the entire network and fine-tuning the last block three times. In particular, these improvements because of using TL, which highly parameterized models are prevented from overfitting and have good generalization, where the most significant benefit of the proposed technique over DL algorithms is that it saves time and hardware costs. Moreover, there is obvious that TL-based models outperform CNN models that are trained from scratch with additional custom layers. The obtained results are presented in Table 5, Table 6 and Table 7, with the AUC values and confusion matrix obtained from each model in Figure 14, Figure 15 and Figure 16.

Furthermore, for the results obtained by DHL-II, one can notice a similar behavior concerning the analysis performed on all models on the OAI dataset for classifying knee OA in different categories, which produced accuracy scores ranging from 87% to 91%.

So far, numerous beneficial research projects have been conducted on the issue that has mostly faced the difficulty of building a model that entirely recognizes KL0, KL1, and KL2, particularly on KL grade 1 because of the slight variations and it is considered to be the beginning of the disease. Therefore, they resorted to grouping KL grades to evaluate their algorithms. However, according to our research, it would be more appropriate to classify KL grades as continuous variables based on their severity, resulting in more accurate forecasts of disease progression. In Table 8 and Table 9, which demonstrate the proposed DHL’s execution time and accuracy, we can observe a benefit from the two proposed DHL models in time, accuracy, and hardware costs.

#### 4.3.1. The Performance of the Proposed DHL-II for Classifying Knee OA Using Four Classes

In this experiment, we will classify particular knee x-ray images into four classes by grouping the KL grades of the OAI dataset. KL0 and KL1 will represent “No OA,” while KL2 to KL4 will represent mild, moderate, and severe OA, as recommended by [20]. As a result of the grouping of the dataset, we utilized the proposed DHL-II approach to examine our approach’s performance in classifying knee OA into four categories. Experimental results of the proposed DHL-II are presented in Table 5 and Figure 15. In terms of selecting performance metrics for evaluating the proposed DHL-II model for classifying knee OA into four classes, it achieved an 88% accuracy rate, 87.8% average recall, 89% average precision, 94.5% average specificity, and 88.5% average F1-score. The results reported in Table 5 and Figure 14 indicate that the proposed DHL-II achieved the highest accuracy and AUC in comparison with Table 4 and Figure 13, which classify knee OA into five classes.

#### 4.3.2. The Performance of the Proposed DHL-II for Classifying Knee OA Using Three Classes

According to [3], we also performed further evaluations in three classes of the OAI dataset by grouping KL0 and KL1 into a “No OA” group, KL2 into an “Early OA” group, and KL3 and KL4 into a “Severe OA” group. Following that, the dataset was provided to the proposed DHL-II for classification into three classes of knee OA. As a result of our proposed DHL-II model, we were able to demonstrate an accuracy rate of 87%, an average recall of 85%, an average precision of 87.7%, an average specificity of 91.7%, and an 86% average F1-score. Detailed comparisons of sensitivity, specificity, and F1-score are indicated in Table 6. In Figure 15, you can see the AUCs, ROC curves, and confusion matrix of the proposed DHL-II on three classes.

#### 4.3.3. The Performance of the Proposed DHL-II for Classifying Knee OA Using Two Classes

In this experiment, the study was primarily focused on binary classification, whether the particular image was infected with OA or not. As a result, we modified the OAI strata, where KL0 and KL1 were denoted as “Normal”, while KL1 to KL4 were grouped as “Abnormal”, as proposed in [22]. The proposed DHL-II was applied to the changed dataset. Accordingly, the accuracy rate, average recall, average precision, average specificities, and the average F1-score for the study achieved 90.8%, 90 %, 91%, 90.8%, and 90.5%, respectively. A detailed comparison of common performance measures, including sensitivity, F-score, and specificity are presented in Table 7. Figure 16 also includes the ROC curves, AUCs, and confusion matrix for the proposed DHL-II.

### 4.4. Comparison with Previous Works

There has been a significant increase in the use of deep learning to estimate the severity of OA in recent years. Hence, we evaluated our proposed model in comparison to the existing state-of-the-art. Table 9 shows an overview of the studies conducted using the same dataset in testing to classify. In [19,21], CNN-based approaches for detecting knee disease are presented. The study by [19] played a pioneering role in this field, while the approach by [21] produced the most recent new results in the KL classification, where they achieved 61.9% and 66.68% accuracies. However, the VGG-19 network-based approach that has been presented in [1] outperformed this, achieving 69.70% accuracy. In contrast, the author in [20] further increased the sensitivity and specificity of the model to 77.2% and 91.5% using Ensemble Learning of DenseNets, apart from grouping the KL0 and KL1 into a challenging class due to the small variations, while KL2 to KL4 classes remain the same. In addition, [5] improved OA severity classification performance by using the TL in the object detection domain and attention technique. In contrast, [23] uses deep convolutional networks by using fine-tuned pre-trained ResNet-169 TL for classification rather than mentioning the detection procedure. Similar to the study in [20], the authors in [3,22] have also carried out a binary classification using deep neural networks and achieved optimum performance with an accuracy of 79%, whereas in [3], they classified knee OA severity into three classes using DeepCNN and leverages an ensemble network of 50 layers.

From Table 9, the comparison of our experimental results with the state-of-the-art, we find that all existing algorithms are less robust and classify knee disease with less accuracy than both our proposed DHL models. Accordingly, the DHL models have improved sensitivity, accuracy, and specificity compared with other approaches. The comprehensive experimental results of the proposed CNN models are presented in detail throughout Table 3, Table 4, Table 5, Table 6 and Table 7, as well as Figure 12, Figure 13, Figure 14, Figure 15 and Figure 16.

## 5. Discussion and Conclusions

In this paper, an efficient method for diagnosing and classifying knee OA severity based on X-ray images has been proposed, which employs pre-trained CNNs for feature extraction as well as fine-tuning the pre-trained CNN using the TL method. In this regard, two DHL (DHL-I and DHL-II) models were created. The first DHL-I approach was based on pre-trained CNNs, PCAs, and SVMs for classifying knee OA severity. Through the pre-trained CNN model, the proposed DHL-I model allows for exploiting the ability of OA to generate diverse features from X-ray images. By incorporating these features into SVMs, we achieved excellent generalization abilities and higher classification accuracy than the previous methods. Experiments were conducted on an OAI dataset to test the performance of the proposed model. Experimental results show that the proposed DHL-I model achieved high accuracy levels both in training and testing data. Further improvements to OA severity diagnostic accuracy were made to classify knee OA into different class labels by testing TL with a pre-trained CNN to see if it handled overfitting and time complexity problems based on the proposed DHL-II model, which showed an improvement in the performance of the model when compared to the related researches. However, the DHL-II shows that the features from the pooling and convolutional layers are more accurate than those from the FC layers. Therefore, fine-tuning networks used to involve replacing the top FC layer, resulting in better classification accuracy. However, there are several limitations faced by our study. Firstly, in the OAI dataset study, skyline view radiographs were not acquired, which would have provided further discriminative information. Therefore, adding lateral view images can be helpful when examining structural features and provide additional information about the patellofemoral joint and femoral osteophytes, which are not visible from PA radiographs alone. Secondly, the proposed models include knee OA radiographs of both tibial condyles and the femoral crista and articulation of both medial and lateral patellofemoral joints, but this requires time. Despite this limitation, this would give the model more information about the relationship between bones in the knee joint. Thirdly, classifying Knee OA images on KL grade 1 is challenging because of the small variations, particularly between grades 0 to grade 2, thus needing powerful feature extraction to address this problem. The final and main limitation of this work is a common problem with ML for medical applications is class imbalance. Therefore, we often have to deal with datasets where one of the classes is significantly under-represented. Consequently, the classification problem becomes harder for the model, and it risks detecting the minority class incorrectly.

Despite the mentioned limitations above, the experimental results of the proposed two DHL models outperformed recent methods compared to the previous work in the literature. To conclude, utilizing the suggested DHL framework, rapid and computer-assisted diagnosis may contribute to early classification in the first stage of the disease to help reduce its progression and improve people’s quality of life.

## Figures and Tables

**Table 1:** KL grades frequency distribution among training, validation, and testing process.

Set\ KL Grades	0	1	2	3	4
**Train**	2777	1274	1856	926	212
**Valid**	308	142	206	103	24
**Test**	772	354	516	257	59

**Table 2:** The configuration detail of the proposed model with their output size.

Layer Name	Output Size	Layer Information
Input Layer	112 × 112 × 1	
conv2_d(1 block)	112 × 112 × 32	3 × 3, 32, padding same
conv2_d(1 block)	112 × 112 × 32	3 × 3, 32, padding same
conv2_d(1 block)	112 × 112 × 32	3 × 3, 32, padding same
max_pooling2_d	56 × 56 × 32	2 × 2, dropout = 0.25
conv2_d_(2 block)	56 × 56 × 64	3 × 3, 64, padding same
average_pooling2_d	28 × 28 × 64	2 × 2, dropout = 0.5
conv2_d_(3 block)	28 × 28 × 128	3 × 3, 128, padding same
average_pooling2_d	14 × 14 × 128	2 × 2, dropout = 0.75
flatten	25088	
fully connected layer	200	dropout = 0.5
Softmax	Output five classes	0,1,2,3,4
**Total parameter**	**5,129,973**	

**Table 3:** Experimental results of the proposed CNN trained from scratch on testing data.

Grade	Recall (%)	Precision (%)	Specificity (%)	F1-Score (%)
0	87	63	66	73
1	12	33	94	18
2	53	59	86.9	56
3	72	76	96.5	74
4	69	85	99.6	77

**Table 4:** The test set performance of our proposed DHL-I applied to the unseen dataset.

Grade	Recall (%)	Precision (%)	Specificity (%)	F1-Score (%)
0	88	75	80.5	81
1	27	64	96.7	38
2	77	70	88.4	74
3	90	85	97.7	88
4	95	90	99.7	93

**Table 5:** The obtained experimental results when the proposed DHL-II is implemented on the unseen dataset of four classes.

Grade	Recall (%)	Precision (%)	Specificity (%)	F1-Score (%)
0	94	90	85	92
1	75	82	94	78
2	87	92	99	90
3	95	93	100	94

**Table 6:** Performance of the proposed DHL-II applied to the unseen dataset of three classes.

Grade	Recall (%)	Precision (%)	Specificity (%)	F1-Score (%)
0	93	88	83	91
1	71	80	93	75
2	92	95	99	93

**Table 7:** Results of our proposed DHL-II applied on the unseen dataset of two classes.

Grade	Recall (%)	Precision (%)	Specificity (%)	F1-Score (%)
0	93	91	90.8	92
1	87	91	90. 8	89

**Table 8:** Execution time of the two proposed DHL models using pre-trained CNN.

Methods	Total Training Time
Proposed CNN Proposed DHL-I	~7.23 h ~11.64 min
Proposed DHL-II for 4 classes	~8.25 min
Proposed DHL-II for 3 classes	~8.61 min
Proposed DHL-II for 2 classes	~ 7.60 min

**Table 9:** Evaluation of the proposed method with other existing methods in terms of recall, specificity, and accuracy.

Method	Year	Dataset	*n* Classes	Recall (%)	Specificity (%)	Accuracy (%)
CNN [19]	2017	MOST + OAI	5 classes	62	-	61.9
DenseNets(ensemble) [20]	2018	OAI	4 classes	77.2	91.5	78.37
Siamese CNNs [21]	2018	MOST + OAI	5 classes	-	-	66.71
Pretrained VGG-19 [1]	2019	OAI	5 classes	-	-	69.70
ML [24]	2019	OAI	2 classes	82.15	80.65	82.98
ResNet-169 (TL) [23]	2020	OAI	5 classes	69.6	-	70.66
DNN [22]	2020	OAI	2 classes	-	-	up to 79.6
DeepCNN [3]	2021	MOST + OAI	3 classes	-	-	73.46
DeepCNN [3]	2021	MOST + OAI	5 classes	-	-	66.68
Attention with CNN [5]	2021	OAI	5 classes	-	-	69.18
**Proposed CNN**		**OAI**	**5 classes**	**59**	**63.6**	**62**
**Proposed DHL-I**		**OAI**	**5 classes**	**75.4**	**92.58**	**74.57**
**Proposed DHL II**		**OAI**	**4 classes**	**87.8**	**94.5**	**88**
**Proposed DHL-II**		**OAI**	**3 classes**	**85**	**91.7**	**87**
**Proposed DHL-II**		**OAI**	**2 classes**	**90**	**91**	**90.8**

## Data Availability

Not applicable.
